# The Adverse Effect of Hypertension in the Treatment of Thyroid Cancer with Multi-Kinase Inhibitors

**DOI:** 10.3390/ijms18030625

**Published:** 2017-03-14

**Authors:** Ole Vincent Ancker, Markus Wehland, Johann Bauer, Manfred Infanger, Daniela Grimm

**Affiliations:** 1 Department of Biomedicine, Aarhus University, Wilhelm Meyers Allé 4, 8000 Aarhus C, Denmark; ole.vincent.ancker@post.au.dk; 2 Clinic and Policlinic for Plastic, Aesthetic and Hand Surgery, Otto von Guericke University, Leipziger Str. 44, 39120 Magdeburg, Germany; markus.wehland@med.ovgu.de (M.W.); manfred.infanger@med.ovgu.de (M.I.); 3 Max-Planck-Institute for Biochemistry, Am Klopferspitz 18, 82152 Martinsried, Germany; jbauer@biochem.mpg.de

**Keywords:** thyroid cancer, hypertension, vascular endothelial growth factor, multi-kinase inhibitors, lenvatinib, sorafenib, sunitinib

## Abstract

The treatment of thyroid cancer has promising prospects, mostly through the use of surgical or radioactive iodine therapy. However, some thyroid cancers, such as progressive radioactive iodine-refractory differentiated thyroid carcinoma, are not remediable with conventional types of treatment. In these cases, a treatment regimen with multi-kinase inhibitors is advisable. Unfortunately, clinical trials have shown a large number of patients, treated with multi-kinase inhibitors, being adversely affected by hypertension. This means that treatment of thyroid cancer with multi-kinase inhibitors prolongs progression-free and overall survival of patients, but a large number of patients experience hypertension as an adverse effect of the treatment. Whether the prolonged lifetime is sufficient to develop sequelae from hypertension is unclear, but late-stage cancer patients often have additional diseases, which can be complicated by the presence of hypertension. Since the exact mechanisms of the rise of hypertension in these patients are still unknown, the only available strategy is treating the symptoms. More studies determining the pathogenesis of hypertension as a side effect to cancer treatment as well as outcomes of dose management of cancer drugs are necessary to improve future therapy options for hypertension as an adverse effect to cancer therapy with multi-kinase inhibitors.

## 1. Introduction

The most common and effective strategies to treat thyroid cancer are surgery, radioactive iodine (RAI) therapy and thyroid-stimulating hormone (TSH) suppression treatment. This therapy regimen shows good results in patients affected by differentiated thyroid carcinoma (DTC) as well as a long-term survival rate of up to 90% [1]. The therapy options for de-differentiated thyroid cancers or for recurrent thyroid cancer are extremely limited. Poorly differentiated thyroid cancer types (PDTC) do not respond to RAI treatment and have a remarkably reduced survival rate. Under these circumstances, multi-kinase inhibitors, such as lenvatinib, sorafenib and sunitinib, may be useful. The multi-kinase inhibitors target an important step in the development of tumors. When a tumor reaches a critical level in its development, oxygen must be delivered through blood vessels and not simply by diffusion. At this point, the tumor produces new blood vessels and thereby obtains the required oxygen and nutrition to grow. The multi-kinase inhibitors work anti-angiogenically by preventing the transmission of signals from multiple tyrosine kinases, which are essential for the development of a new vasculature [2].

Along with their effects as cancer drugs, multi-kinase inhibitors have been shown to cause several unwanted side effects; examples are proteinuria, stomatitis, diarrhea and hypertension, the latter of which had been observed in up to half of the treated patients [3].

Hypertension, or elevated blood pressure, is a physical condition in which the pressure in the blood vessels is persistently raised and the heart must labor against higher systolic and/or higher diastolic pressure. Hypertension exists per definition when the systolic blood pressure (SBP) equals or exceeds 140 mmHg and/or the diastolic pressure (DBP) equals or exceeds 90 mmHg, whereas normal blood pressure is defined as 120 mmHg systolic and 80 mmHg diastolic [4].

Hypertension can physically be described by Ohm’s law:

blood pressure = cardiac output × total periphery resistance

Isolated hypertension, when not extremely elevated, is not dangerous and many people live with raised blood pressure without even being aware of it. However, hypertension can have severe impacts on overall health, numerous studies have shown that patients with hypertension have a higher risk of cardiovascular and renal diseases [5].

The aim of this review is to create an overview of hypertension as an adverse effect (AE) of multi-kinase inhibitors when treating metastatic RAI-refractory thyroid cancer. In addition, this review will focus on the function of multi-kinase inhibitors, and on the mechanisms of the development of hypertension. It will reflect the importance of hypertension as an AE.

This review will consider and address the following questions: (1) How do multi-kinase inhibitors cause hypertension? (2) How can we manage hypertension induced by tyrosine kinase inhibitor (TKI)-treatment? (3) Is the relationship between the efficacy of cancer treatment and the AE of hypertension favorable? (4) Is hypertension as a side effect of the multi-kinase inhibitors a severe concern?

## 2. Background

### 2.1. Thyroid Cancer

The thyroid gland is located in front of the tracheal tube. The function of the thyroid gland is to produce the thyroid hormones T3 and T4, which stimulate a great number of processes in the human body, such as metabolic rate, protein synthesis, development, and they also influence the cardiovascular system. Furthermore, the thyroid produces calcitonin, which plays a role in calcium homeostasis. The thyroid gland can be enlarged both by benign and malignant causes: it is often enlarged due to a dietary iodine deficiency that is not cancer associated (struma), but other tumors of the thyroid are caused by malignant alterations [6]. Thyroid cancer can be classified into several categories: differentiated (DTC), covering papillary (PTC) and follicular (FTC), medullary (MTC) and anaplastic thyroid cancer (ATC). The cancer cells in DTC appear similar to normal thyroid cells, whereas poorly differentiated thyroid cancer (PDTC) is comprised of cancer cells that do not share the same characteristics or abilities as normal thyroid cells [7].

PDTC is accountable for up to 10% of thyroid cancer forms and is more aggressive than DTC. Unfortunately, the prognosis for PDTC is not encouraging: the 5-, 10- and 15-year survival rates are 50%, 34% and 0%, respectively [8]. MTC has a 10-year survival rate of 75%–80% [9], whereas the 5-year survival rate for DTC is as high as 98% due to successful treatment, such as surgery, RAI ablation and treatment with thyroid stimulating hormone [10]. Regrettably, about 20% of patients experience recurrence of the disease. The recurrent form of thyroid cancer is poorly differentiated and thereby more malignant; this makes it for example resistant to RAI ablation because due to an inability to take up iodine [11].

The incidence of thyroid cancer in Denmark in 2012 was estimated to be 220 new cases, both sexes included, which places it in the intermediary group below the most common cancer types as colorectal, breast and prostate cancer [12]. In 2012, the incidence worldwide was 298,102 new cases for both sexes representing 2.1% of all cancers [13].

### 2.2. Multi-Kinase Inhibitors

Most thyroid cancer types have promising prospects as a result of surgical treatment and radioactive iodine ablation. PDTC, RAI-refractory carcinomas and tumors showing resistance to various forms of available treatment must be treated with alternatives in the hope of a good result [14].

Angiogenesis is the formation of new blood vessels from pre-existing vasculature and is a normal physiological process that starts during fetal development and persists in adults during inflammation and vascular or wound healing [15]. Angiogenesis is utilized by tumors to create new blood supply, so forming a path for the delivery of oxygen and nutrients, and in turn supporting tumor growth. Several factors play a role in the creation of new blood vessels, both in normal physiology and in pathophysiology. The growth of a tumor is determined by its nutrient supply. By diffusion alone, only very limited amounts can reach the tumor, and especially its core, so that for continued expansion, an increased internal blood supply is necessary. The central hypothesis is that an increase in tumor size must be preceded by expansion of tumor vasculature, which is stimulated by the tumor. Tumor cells take advantage of the normal physiological process involving the secretion of vascular endothelial growth factor (VEGF). VEGF is of high importance in the induction of new vessel formation and in the survival of endothelial cells [16,17,18]. Therefore, angiogenesis is a critical process for the development and subsequent growth of tumors.

The superfamily of VEGF comprises VEGF-A, VEGF-B, VEGF-C, VEGF-D and placental growth factor (PGF). Their corresponding tyrosine kinase receptors, the vascular endothelial growth factor receptors (VEGFRs) VEGFR-1, -2 and -3, are distinguished according to their affinities to different VEGFs. VEGFR-1 and -2 are found in endothelial cells, while VEGFR-3 is expressed in lymphatic endothelial cells. VEGFR-1 binds VEGF-A, VEGF-B and PGF, whereas VEGFR-2 binds VEGF-A and proteolytically modified VEGF-C and VEGF-D. Finally, VEGFR-3 is activated by VEGF-C and VEGF-D [19,20,21].

In addition, growth factors platelet derived growth factor (PDGF), epidermal growth factor (EGF), and fibroblast growth factor (FGF) also play a significant role in angiogenesis [19,20]. The ligand FGF and its receptor FGFR play an important role in cell growth, proliferation, differentiation and survival of thyroid cancer cells, where FGFR-1, -3 and -4 are overexpressed and expression of FGFR-2 is reduced. Binding of a growth factor to one of the receptors leads to a tyrosine kinase activation of either the mitogen activated protein kinase (MAPK) or the phosphatidylinositol-3-kinase (PI3K) pathway that eventually affect oncogenic gene expression [22].

There are two main pathways by which VEGF signaling can be interfered pharmacologically. Direct inhibition of VEGF is one possibility: an immunoglobulin designed specifically for VEGF targets and binds before the interaction with the corresponding receptor [23]. Another pathway focuses on inhibition of the phosphorylation cascade triggered after the binding of ligand and receptor by blocking the signal from the tyrosine receptor and thereby preventing the oncogenic features such as angiogenesis, proliferation and growth. Lenvatinib, sorafenib and sunitinib (Table 1) are drugs used in cancer therapy that inhibit multiple tyrosine kinases in thyroid cancer treatment [22].

**Figure 1:**
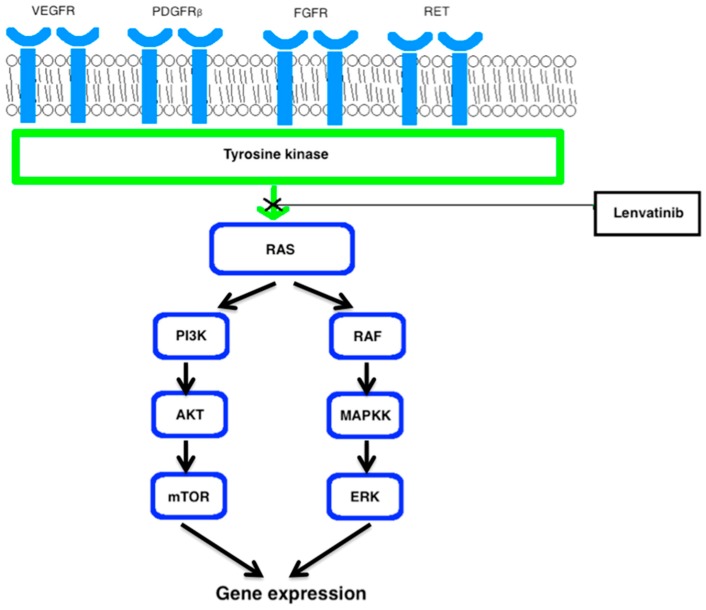
Lenvatinib inhibits signaling from VEGFR, PDGFRβ, FGFR and RET. It decreases angiogenesis and lymphogenesis, stunts tumor growth and damages the tumor’s microenvironment [11,22]. VEGFR (vascular endothelial growth factor receptor), PDGFR (platelet derived growth factor receptor), FGFR (fibroblast growth factor receptor), RET (rearranged during transfection), RAS (rat sarcoma), PI3K (phosphatidylinositol-3-kinase), AKT (protein kinase B), mTOR (mammalian target of rapamycin), RAF (rapidly accelerated fibrosarcoma kinase), MAPKK (mitogen activated protein kinase kinase), ERK (extracellular signal regulated kinase).

Lenvatinib is an orally taken TKI that targets VEGF-R1/-3, FGFR1-4, ret proto-oncogene (RET), and platelet derived growth factor receptor (PDGFRβ). By blocking these receptors, lenvatinib disturbs angiogenesis of the tumor, invasion of tissue and metastasis. By inhibiting VEGF-R1/-3, lenvatinib disrupts angiogenic processes in the tumor. Furthermore, lenvatinib inhibits RET, which is important for controlling tumor growth and, by hitting FGFR and PDGFRβ, it also influences the tumor’s microenvironment, as presented in Figure 1 [22].

Sunitinib (Sunitinib malate; Sutent; Pfizer, New York, USA) is a multi-targeted TKI used in the treatment of metastatic renal cell carcinomas (RCC) and gastrointestinal stromal tumors, and is under evaluation for other malignancies [11]. It inhibits VEGFR-1 and -2, platelet-derived growth factor receptors, stem cell factor receptor (c-KIT), FLT3 as well as RET kinases. Sunitinib acts on VEGF receptors and on RET and is therefore a suitable drug to treat RAI-refractory thyroid cancer. Sunitinib is still not approved for thyroid cancer therapy by the FDA, and therefore therapy approaches applying sunitinib are still off-label [11].

Sorafenib (NEXAVAR^®^) is widely used in cancer therapy and is an oral serine-threonine TKI that targets VEGFR-1/–3, PDGFR, BRAF, RET/PTC, and c-kit. The agent has an anti-proliferative effect and an anti-angiogenic activity by blocking the intracellular signal transduction of VEGFR2 in endothelial cells [11]. Sorafenib is a FDA-approved drug for patients with RAI-refractory metastatic thyroid cancer. The approval of sorafenib is based on the results of the randomized DECISION (stuDy of sorafEnib in loCally advanced or metastatIc patientS with radioactive Iodine refractory thyrOid caNcer) trial. This was an international, multi-center, placebo-controlled study involving 417 thyroid cancer patients. The patients (400 mg oral sorafenib twice daily) lived on average nearly 11 months longer without disease progression compared to the placebo group [24,25,26].

### 2.3. Hypertension

**Figure 2:**
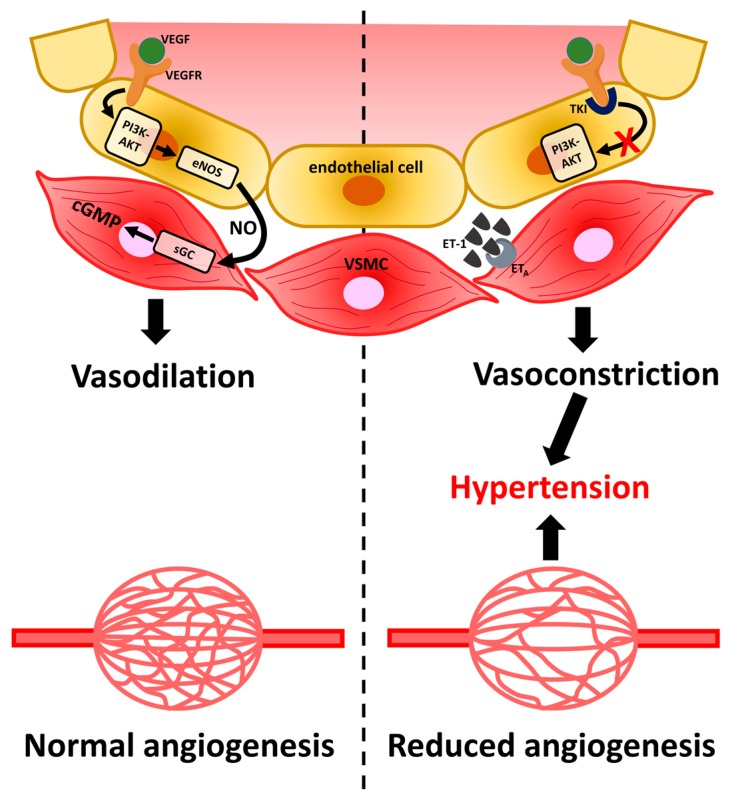
The effects of VEGF on blood pressure and capillary vascularization under: physiological conditions (**left**); and TKI therapy (**right**) (adapted from [39]). VEGF (vascular endothelial growth factor), VEGFR (vascular endothelial growth factor receptor), PI3K (phosphatidylinositol-3-kinase), AKT (protein kinase B), eNOS (endothelial nitric oxide synthase), sGC (soluble guanylyl cyclase), ET-1 (endothelin-1), ET_A_ (endothelin receptor type A), NO (nitric oxide), cGMP (cyclic guanosine monophosphate).

The effects of VEGF are not only present in cancer cells: healthy endothelial cells also express VEGFRs and, because of this property, unwanted consequences may appear [20,27,28]. Hypertension is the most common adverse effect in the treatment of the tyrosine kinase inhibitors. VEGF is known to regulate the vasomotor tonus and maintains blood pressure by dilating small arterioles and venules. In case of an anti-VEGF therapy the result is a reduced density of microvessels (Figure 2). Hypertension has been reported to occur at a higher incidence in patients with DTC and treated with sorafenib [29]. Hypertension usually occurs in the first six weeks of treatment with sorafenib; therefore, blood pressure (BP) should be monitored regularly (at least once a week) at the start of sorafenib therapy [30].

In the SELECT trial (ClinicalTrials.gov number, NCT01321554), lenvatinib, compared to placebo, revealed significant improvements in progression-free survival and the response rate in patients with RAI-refractory thyroid cancer, but it also induced more adverse effects [31]. Treatment-related adverse effects (TEAE) of any grade, occurring in more than 40% of lenvatinib-treated patients were hypertension (in 67.8% of the patients), diarrhea (in 59.4%), fatigue or asthenia (in 59.0%), decreased appetite (in 50.2%), decreased weight (in 46.4%), and nausea (in 41.0%) [31]. Cabanillas et al. [32] investigated 58 patients with advanced, progressive, RAI-refractory DTC, receiving lenvatinib 24 mg once daily in 28-day cycles until disease progression, unmanageable toxicity, withdrawal, or death. TEAE were evaluated: 44 patients had hypertension (all grades 76%) and six patients had grade 3 TEAE (10%). Most patients with hypertension and proteinuria were managed successfully without lenvatinib dose adjustments [32].

There is no unanimous agreement on how these cancer drugs result in hypertension, but some hypotheses have gained a footing in explaining why. One explanation depends on reduced production of the vasodilator, nitric oxide (NO). Blockage of VEGF induces vasoconstriction. VEGFR-2 signaling generates nitric oxide (NO) and prostaglandin I2, which induces endothelial cell-dependent vasodilatation in arterioles and venules. Inhibition of VEGFR-2 signaling reduces NO synthase expression and NO synthesis. Normally, activation of VEGFR-2, by VEGF or shear stress in the vessel walls, induces the PI3K pathway, resulting in an increased production of the vasodilator NO and hence a reduction of peripheral resistance and blood pressure. VEGF inhibition results in an increase in vascular resistance, followed by hypertension. The multi-kinase inhibitors prevent the phosphorylation cascade and thus the formation of NO, leading to a rise in blood pressure [33]. In addition, an increase in blood pressure may also result from the VEGF/VEGFR inhibition in the kidney. VEGF and VEGFR are also expressed in podocytes. Electron microscopy images revealed glomerular lesions associated with VEGF-targeted therapies [34]. The reduced VEGF activity influences renal endothelial cells and podocytes and results in a dysregulation of VEGF expression and a downregulation of tight junction proteins with the consequence of proteinuria. As an alternative explanation to the theory of the lack of NO, an increased amount of the vasoconstrictor endothelin-1 (ET-1) has been suggested. ET-1 binds to its receptors in endothelial cells causing the smooth muscle cells to contract and thus increase resistance in the vessels and raise the blood pressure [35,36].

A study has considered a third theory that suggests hypertension is due to a decrease in the number of small arterioles and capillaries, leading to higher peripheral resistance and thereby to increased blood pressure. The authors found that patients treated with anti-angiogenic medicaments showed fewer mucosal capillaries. However, the study was not able to determine whether the observed effects were a consequence of a direct lack of small arterioles or simply a hypo-perfusion of these, since the technique used for recording the numbers of vessels depended on perfusion. Both a decreased number of arterioles or a stopping of perfusion of existing ones could explain a rise in blood pressure, since the blood is distributed in fewer vessels, increasing resistance inside them [37].

Some studies have shown a rapid increase in blood pressure, which challenges the understanding of the mechanisms giving rise to hypertension, and argues against a structural or anatomical explanation of acute induced hypertension. However, rapid rises of hypertension make a theory of active vasomodulators more likely [38]. Risk factors for hypertension occurring upon TKI therapy are of older age, obesity, high sodium intake, alcohol abuse, smoking or reduced physical activity. A pre-existing high blood pressure or certain VEGF polymorphisms might be associated with a lower risk of grade 3 or 4 hypertension.

### 2.4. Efficacy of Cancer Drug Treatment

A recent study [31] has monitored the efficacy of lenvatinib, compared with placebo, in adults with DTC, RAI-refractory cancer and without prior treatment with a multi-kinase inhibitor. The primary endpoints were either progression of the cancer disease or death. Patients treated with lenvatinib had a median progression-free survival of 18.3 months, whereas placebo-treated patients had a progression-free survival of only 3.6 months. The six month progression-free survival rate for the patients treated with lenvatinib was 77.5%, while the rate for placebo-treated patients was 25.4%. Patients receiving lenvatinib also had more AE. Treatment-related AE of any grade occurred in more than 40% of patients in the lenvatinib group; for example, hypertension (in 67.8% of the patients), diarrhea (in 59.4%), and others [31].

Another study published in December 2015 showed similar results: Japanese patients with DTC, RAI-refractory disease and a progression in disease in the last 13 months were eligible. Patients treated with lenvatinib experienced a median progression-free survival of 16.5 months, while the placebo group had a progression-free survival of 3.7 months. The six-month progression-free survival rate was 70.0% for lenvatinib-treated patients and 31.7% for placebo-treated patients [40]. The most common AE (any grade) in Japanese patients from the SELECT trial was hypertension (86.7%).

Taken together, the findings of the SELECT trial provided the basis for the FDA approval of lenvatinib for the treatment of progressive RAI-refractory thyroid cancer. Another study investigated 59 patients with unresectable progressive MTC [41]. They received lenvatinib (24 mg daily, 28-day cycles) until disease progression, unmanageable toxicity, withdrawal, or death. Lenvatinib had a high objective response rate, a high disease control rate, and a short median time to response. Toxicities were managed with dose modifications and medications. Most hypertension and proteinuria events were grade 1 or 2 and managed with standard drug therapy. The median duration of treatment was 264 days (range, 13–547 days). Withdrawal from therapy due to hypertension: one patient (2%).

Hypertension is a condition that affects the cardiovascular system in the body. It is an abstract condition because not all patients have the same tolerance of raised pressure. Some may experience serious cardiovascular outcomes, while others can live their whole life with hypertension without showing any symptoms. Studies show that hypertension is associated with many different cardiovascular diseases, where almost all have a high mortality rate, such as intracerebral hemorrhage, subarachnoid hemorrhage, stable angina pectoris, myocardial infarction, aorta aneurysm and heart failure [42].

Most patients with hypertension as an adverse effect continue the treatment of cancer. In a lenvatinib-versus-placebo study, only 1.1% of patients had to stop the treatment because of hypertensive effects and 19.9% were given a lower dose because of a rise in BP [31]. Nearly all patients with this form of hypertension manage the symptoms with normal anti-hypertensive drugs. Patients with unmanageable hypertension and signs of organ damage, renal dysfunction or cardiovascular diseases need intervention in the form of either a lower dose or a total stop of treatment [38]. Hypertension, and other AE from TKI-treatment are currently under investigation. Several clinical trials are investigating this concern, both examining the direct AE of lenvatinib, but also whether lower doses of the drug can show the same effect while giving fewer unwanted effects. An overview of recent studies is given in Table 2.

The study NCT02915172 includes both a phase I and a phase II study. The phase I study aims to decide the highest tolerable dose of lenvatinib and capecitabine in patients with advanced cancer. The initial dose for the first included group is 10 mg orally taken lenvatinib in a 21-day cycle. The phase II study wishes to determine whether the maximal tolerated dose found in the phase I study can be used for treating patients with advanced cancer including thyroid cancer. NCT02915172 takes place at the MD Anderson Cancer Center, Texas, USA, which is also the responsible party. The study will include 46 participants over the age of 18. The study was set to start December 2016 and the final data collection date is set to December 2022.

NCT02430714 and NCT02764554 are post-marketing surveillance studies of lenvatinib in patients with unresectable thyroid cancer. In NCT02430714, a dose of 24 mg orally taken once daily is administered to the patients and, in NCT02764554, the participants are in groups with 4 mg and 10 mg orally taken lenvatinib. The primary outcome is to decide the number of AE in the following year. NCT02430714 is conducted by the Japanese pharmaceutical company Eisai Co., Ltd., Japan, recruiting 400 participants including children, adults and seniors from centers in Osaka and Tokyo, Japan. The study was set to start May 2015 and the final data collection date is March 2025. NCT02764554 is performed by the Korean pharmaceutical company Eisai Korea Inc. The estimated number of enrolled patients is 3000 including children, adults and seniors recruiting from Seoul, Republic of Korea. The start date of the surveillance study was September 2016 with a final data collection date of July 2021.

The study NCT02702388 investigates whether a lower dose, than the currently approved dose of 24 mg orally taken lenvatinib once daily, shows a comparable effect but a better safety profile in patients with RAI-refractory DTC. The two experimental starting doses are 20 mg and 14 mg reducing down to a dose of 4 mg. NCT02702388 includes 41 participants over the age of 18. The start date was set to March 2016 and the final data collection date is May 2019. The 78 centers of the study are located in Australia, Austria, Belgium, Denmark, France, Germany, Italy, Philippines, Poland, Portugal, Romania, Spain, Sweden, United Kingdom and United States of America. The responsible party of the study is Eisai Inc.

The aim of the studies NCT00280397 (solid tumors) and NCT00121719 (solid tumors) is to investigate the maximal tolerated dose of lenvatinib and furthermore, to provide a summary of AE and the pharmacokinetic properties of lenvatinib. NCT00280397 started in January 2006 and had its final data collection date in September 2008. The study consisted of 27 participants with an age span from 20 to 75 years. The study was performed by Eisai Inc. with a center in Tokyo, Japan. The study found the maximal tolerated dose of lenvatinib to be 13 mg, when orally taken two times daily two-weeks-on/one-week-off [44]. NCT00121719 began in July 2005 with a final data collection date in June 2009. Eighty-seven participants over the age of 18 were enrolled in the study. The study was conducted by Eisai Inc. with centers in Amsterdam, Netherlands and Glasgow, United Kingdom. The study found the maximal tolerated dose of lenvatinib to be 25 mg orally taken once daily.

The studies NCT01728623, NCT02726503, NCT00784303, and NCT02966093 intend to determine the overall- and progression-free survival rates of participants treated with 24 mg lenvatinib orally taken once daily, moreover the studies aim to investigate the AE of the drug. NCT02726503 also studies the efficacy on ATC (Phase II) and NCT00784303 furthermore investigates the tumor response rate and the pharmacokinetic profile of lenvatinib. NCT01728623 started September 2012 and had its last data collection date in July 2015. In this study 37 participants over the age of 20 years were enrolled from three centers in Japan. The responsible party of the study was Eisai Inc. NCT02726503 started in January 2016 and has its last data collection date in July 2018. The study consists of 39 participants over the age of 20 years recruiting from 12 Japanese hospitals. The responsible party of this study is the Translational Research Informatics Center, Kobe, Hyogo, Japan. NCT00784303 started in August 2009 and had its last data collection date in April 2011. One hundred sixteen participants over the age of 18 were enrolled from 51 centers in the United States, Australia, France, Germany, Italy, Poland and the United Kingdom. The responsible party is Eisai Inc. NCT02966093 is set to start in January 2017 and has its final data collection date in January 2020. The estimated number of participants is 150 over the age of 18. Recruiting from 13 centers in China. Eisai Co., Ltd. is the responsible party of this study.

Study NCT02185560 aims to analyze patients of all ages and sexes, suffering from unresectable differentiated thyroid carcinoma and receiving sorafenib treatment. Standard follow-up length will be 9 months (or until lost to follow-up). For patients with a possible follow-up of 24 months, additional data, such as survival status and keratoacanthoma and/or squamous cell cancer development will be collected. Primary end points are the number of participants with adverse and serious adverse drug reactions as well as the number of participants with serious AE as a measure of safety and tolerability. This study is conducted by Bayer Clinical Trials.

The Phase 2 study NCT02084732, conducted by the Instituto Nacional de Cancerologia, Columbia, is also directed towards an assessment of the safety and efficacy of sorafenib in the treatment of patients with advanced thyroid cancer. It is currently in the recruiting stage and is open for adult patients of both sexes. The follow-up period is 24 months, and besides its primary objective of determining the clinical activity and safety profile of sorafenib in the treatment of patients with advanced thyroid cancer, its secondary aims are the measurement of PFS and the description of AE associated with sorafenib used in advanced thyroid cancer.

Study NCT02185560 is similar in design and scope to NCT02185560 discussed above, just substituting sorafenib with sunitinib. Bayer Clinical Trials is the responsible party.

Study NCT00510640 has been conducted by the University Hospital Bordeaux, France under collaboration with Pfizer. Patients suffering from locally advanced or metastatic anaplastic, differentiated or medullary thyroid carcinoma receive 50 mg/day sunitinib for four weeks followed by a rest-period of two weeks. Cycles are repeated until disease progression or severe toxicity. Based on tolerability, doses can be reduced to 12.5 mg/day. Adult patients of both sexes are eligible. The primary outcome measure of this study is the objective response rate (ORR) (the proportion of patients with confirmed complete (CR) or partial response (PR) according to the RECIST), secondary outcome measures are the safety of sunitinib in patients with thyroid carcinoma and time to disease progression, response, and duration of response. The study is completed, and no results are published so far.

In the study NCT00668811 a total of 23 patients with advanced differentiated thyroid cancer received 37.5 mg/day sunitinib for treatment cycles of 28 days. Adult patients with advanced DTC were eligible and of the 23 subjects, 74% (17) were male. The primary objective is to assess clinical benefit rate, defined as complete response, partial response, or stable disease and the secondary objective is to assess the safety of sunitinib in this patient population. Overall, this study found, that sunitinib was relatively well tolerated and had good anti-tumor activity for this kind of thyroid cancer [45]. The responsible party for this study was the Washington Hospital Center in collaboration with the company Pfizer.

For an overview of adverse effects observed in larger clinical trials with lenvatinib, sofrafenib, and sunitinib, see Table 3.

### 2.5. Management of Multi-Kinase Inhibitor-Caused Hypertension

It is important to measure baseline BP in order to better determine cardiovascular risk factors after administering treatment. If the BP < 120/80 mmHg, TKI therapy can begin [47]. BP monitoring under TKI therapy should be performed every week for the first eight weeks, and before any infusion or cycle. When patients show prehypertension values (120 < SBP < 140 mmHg and 80 < DBP < 90 mmHg), it is important to search for cardiovascular risk factors. If no risk factors are present, TKI treatment can begin and BP should be controlled accordingly. In cases when cardiovascular risk factors are detectable, an antihypertensive therapy, for example, calcium channel blockers (CCB), should be started 3–7 days before the start of TKI therapy.

Nebivolol is a β-adrenoceptor antagonist (BAA) whose antihypertensive effect is mainly related to a reduction in peripheral resistance; therefore, this is a good candidate for treating the TKI-induced rise in BP [48]. However, it is important to consider each patient individually, as there is no golden standard for treatment of TKI-induced hypertension and because every patient is diverse in his or her anamnesis. Some patients may take medicine that affects the metabolism of the antihypertensive or the anti-angiogenic drug and an individual plan must be made for each patient. For example, the multi-kinase inhibitor sunitinib affects the CYP3A4 enzyme, which is of high importance in metabolizing many drugs. This property of sunitinib is important to keep in mind when planning an antihypertensive strategy [49].

Hypertension should be monitored and treated according to standard medical practice, i.e., it should be managed as in patients without cancer. For a patient without comorbidities, the target blood pressure should be <140/90 mmHg. For patients with chronic kidney disease, the target BP is <135/85 mmHg, and for patients with proteinuria, drugs inhibiting the renin-angiotensin system should be applied. The anti-angiogenic therapy should be continued without dose reduction unless severe or persistent hypertension is present [47].

It is difficult to determine which is the best antihypertensive drug because there has been a lack of controlled clinical studies until now. In general, there are several classes of antihypertensive drugs, which can be applied. When a patient exerts signs of proteinuria, angiotensin converting enzyme inhibitors (ACEi) or angiotensin 2 receptor antagonists can be given. In addition, calcium channel blockers such as amlodipine can be considered. Non-dihydropyridine drugs such as verapamil or diltiazem should be avoided because of CYP3A4 metabolism. They are contraindicated in combination with oral angiogenesis inhibitors. In addition, medicaments increasing NO, like nitrates, or the BAA nebivolol can be applied. Common treatments of hypertension involve BAA, CCB, diuretics, ACEi, angiotensin-II receptor antagonists (AIIA), and NO donors [49]. The current treatment of TKI-induced hypertension uses therapeutic options that are already established and is summarized in Table 4. Studies have shown that dihydropyridine CCBs, such as amlodipine, have a great effect on relaxing smooth muscle cells and thereby lowering the total periphery resistance and hence the blood pressure. ACEi and AIIA have also shown good results in the antihypertensive treatment and concurrent renal-protecting effects in these treated cancer patients [39,50].

Finally, based on the proposed mechanisms regarding the lack of NO production, therapeutic considerations may include NO derivatives. Agents that increase NO bioavailability, such as long-acting nitrates are interesting. By giving long lasting precursors of NO, the desired effect is to neutralize or minimize the multi-kinase inhibitor-induced hypertension. In general, NO donors are known to be well tolerated and cause relatively harmless adverse effects such as headache, flushing, nausea and hypotension. Therefore, long lasting NO donors are suggested as a first line treatment [51].

An alternative and newer way to manage TKI-induced hypertension is the application of endothelin receptor antagonists, because it is assumed that ET-1 concentration increases in patients treated with multi-kinase inhibitors. This kind of hypertensive treatment is a novel regimen to handle the AE. Studies have shown that the combination of two or three of the suggested treatments may have a beneficial effect on TKI-induced hypertension [39,52].

### 2.6. Discussion

Hypertension as an AE of multi-kinase inhibitor treatment is an important and frequent concern over TKI treatment. As summarized in the previous sections, numerous AE are occurring with multi-kinase inhibitor treatment of cancer. Grade 1 and grade 2 hypertension, experienced by patients treated with TKI, can frequently be managed without the need for dose reduction or discontinuation of treatment. Hypertension is linked with several cardiovascular and renal diseases, and higher mortality, which make it crucial to investigate [54,55,56,57,58,59].

Not all patients who meet the requirements of the definition of hypertension are affected. Some will never feel any change in their state of health or experience any pathogenic outcomes as a result of hypertension. Meanwhile, other groups of patients suffer from the impact of hypertension consisting of either cardiovascular events or even death. In particular, patients already suffering from risk factors, such as diabetes mellitus, cardiovascular conditions, chronic renal disease or are weakened in other ways are affected [60].

The patients treated with multi-kinase inhibitors, such as lenvatinib and sorafenib, are already in a late stage of their cancer illness and it must be assumed that this group of patients is, in some way, weakened or have sequelae from either the illness itself or from previous treatment. When considering whether hypertension as an AE is critical, it is important to be aware of the individual patient’s anamnesis and general state of health. If the patient shows no previous signs of, for example, cardiovascular events, hypertension usually takes some time to affect the well-being of the patient or to induce life-threatening conditions.

Currently, treatment with multi-kinase inhibitors prolongs progression-free survival by about 12 to 16 months, from a starting point of a couple of months to half a year. Whether this time is enough to develop life-threatening conditions arising from unwanted effects such as hypertension is questionable, but must be considered when evaluating AE. Some above-mentioned studies [31,32] showed that patients either had to stop treatment entirely or lower the dose because of an alarming rise in BP. Other studies suggested the presence of hypertension as a biomarker for a good response to the anti-angiogenic treatment and therefore that hypertension may be necessary, but must also be treated [1,20,32,61]. Similarly, the sunitinib-associated rise in BP, as well as neutropenia, is discussed as biomarkers in metastatic renal cell carcinoma patients: both side effects were associated with longer progression-free survival and a higher overall-survival rate [20]. At the moment, various possible anti-angiogenic biomarkers are under examination, such as hypertension, altered VEGF plasma levels, interleukin (IL)-8 polymorphisms, or a change in tumor microvessel density. Today, promising candidates are detected, but important challenges limit their translation into practice.

Since there is still no generally accepted explanation for the cause of hypertension as an AE in multi-kinase inhibition, a specific therapy to manage this form of hypertension is not given. Various strategies of treating hypertension are suggested, some with good results, but these treatments are only symptomatic and also have accompanying side effects. Therefore, it is important to perform more studies investigating the mechanisms of hypertension induced by TKI, as well as to know the risk factors and the frequency of hypertension induced by anti-angiogenic drugs in different cancer types.

## 3. Conclusions

The multi-kinase inhibition by targeted therapy offers new strategies of treating cancer diseases that otherwise are considered untreatable. Unfortunately, many AE follow this kind of treatment, especially hypertension.

Hypertension is a well-known systemic AE of treatment with VEGF-inhibitors. Treatment induced-hypertension has been associated with sunitinib therapy for different forms of cancer. The TKIs are included in international clinical guidelines as first-line and second-line therapy in metastatic renal cell carcinoma (mRCC). Hypertension is an adverse effect of these drugs and the degree of hypertension associates with the anti-tumor effect in mRCC [62]. More recent phase II trials have shown a significant risk of treatment-induced hypertension with sunitinib in patients suffering from pancreatic neuroendocrine tumors [63] and endometrial carcinoma [64].

In addition, the incidence of treatment-associated hypertension with sorafenib was increased in patients with hepatocellular carcinoma [65] and non-small cell lung cancer [50]. Besides to locally recurrent or metastatic, progressive, RAI-refractory differentiated thyroid cancer, lenvatinib is indicated for the treatment of patients with advanced renal cell carcinoma in combination with everolimus following prior anti-angiogenic therapy. The frequency of hypertension, which is a known class effect of VEGF-targeting agents, was increased in both treatment groups in which lenvatinib was administered [66].

Therapy with multi-kinase inhibitors leads to a relatively large increase in the progression-free survival of patients with late stage metastatic thyroid cancer and not all patients are directly affected by the rise in BP. For some groups of patients with diseases other than thyroid cancer, either simultaneously or previously, hypertension is a great concern that can be dangerous for the individual. Currently, biomarkers for the prediction of the effectiveness of anti-angiogenic therapy are being investigated [67] and in this course, it might be helpful to analyze possible predictors of AE such as hypertension, too. Management of hypertension is somewhat possible, but not with a curative result, and is thereby only symptomatic with the antihypertensive drugs listed in Table 4. Hence, it is important to focus on different regimens for managing high blood pressure, or even to use other strategies to specifically target the cancer cells without causing systemic adverse events.

In summary, the relationship between angiogenic inhibitors and a rise in BP has now been established: angiogenic inhibitors used to treat cancer may exacerbate cardiac risk factors. Introduction or even prophylactic use of antihypertensive drugs can allow maintenance of therapy despite the onset of hypertension. In addition to cancer therapy, the reduction of hypertension risk factors should be addressed.

## 4. Outlook

Several studies have already shown hypertension and other unwanted events in response to multi-kinase inhibition treatment. Future and on-going studies have adjusted their aims to consider the AE of this type of treatment, since many studies with these aims are now registered. It is of great interest and import to examine how to reduce or eliminate AE by dose-finding studies or by determining the mechanisms inducing hypertension and thus being able to properly treat this effect.

## 5. Materials and Methods

Literature and information used for this review can be found using online databases, such as Pubmed, Scopus and clinicaltrials.gov by using the search terms “multi-kinase inhibitors”, “antiangiogenesis”, “multi-kinase inhibitors and hypertension”, “thyroid cancer” and others.

## Figures and Tables

**Table 1:** Characteristics of lenvatinib, sorafenib, and sunitinib.

Drug	Targets	Half-Life	Bioavailability	Metabolism
Lenvatinib	VEGF-R1-3, FGFR1-4, PDGF-RA, c-KIT, RET	28 h	85%	Hepatic CYP3A4
Sorafenib	VEGF-R1-3, PDGF-RA-D, C-RAF, B-RAF	25–48 h	38%–49%	Hepatic CYP3A4
Sunitinib	VEGF-R1-3, PDGF-RA-D, c-KIT, RET, CD114, CD135	40–60 h	50%	Hepatic CYP3A4

**Table 2:** Overview of recent clinical trials studying lenvatinib, sunitinib, and sorafenib, website used on 7 March 2017 [43].

Title	Design	Objective	Status
A phase I trial of lenvatinib (multi-kinase inhibitor) and capecitabine (Antimetabolite) in patients with advanced malignancies. NCT02915172	Interventional open label	This phase I study aims to find the highest tolerable dose of lenvatinib and Capecitabine that can be given to patients with advanced cancer.	Not yet recruiting
Post-marketing surveillance of lenvatinib mesylate in patients with unresectable thyroid cancer. NCT02430714	Observational cohort prospective	The objective of this study is to find unknown adverse reactions, adverse drug reactions, efficacy, safety and effectiveness factors, incidence of hypertension, hemorrhagic, and thromboembolic effects and liver disorder.	Recruiting
A multi center, randomized, double-blind phase II trial of lenvatinib (E7080) in subjects with iodine-131 refractory differentiated thyroid cancer (RR-DTC) to evaluate whether an oral starting dose of 20 mg or 14 mg daily will provide comparable efficacy to a 24 mg starting dose, but have a better safety profile. NCT02702388	Interventional double blind randomized	This randomized double-blinded study aims to investigate whether a lower starting dose of lenvatinib can provide comparable efficacy whilst showing a better safety profile for the patients.	Active Not recruiting
An open label phase I dose escalation study of E7080 administered to patients with solid tumors. NCT00280397	Interventional open label	This study investigates the maximum tolerable dose and the related effects of E7080 (lenvatinib) given to patients with solid tumors with no successful treatment.	Completed
A phase II study of E7080 in subjects with advanced thyroid cancer. NCT01728623	Interventional open label	This study was performed to evaluate the safety, efficacy and pharmacokinetics of E7080 (lenvatinib), taken orally daily in patients with advanced thyroid cancer.	Completed
An open label phase I dose escalation study of E7080. NCT00121719	Interventional open label	This study aims to find the maximum tolerated dose of lenvatinib in patients with solid tumors or lymphomas.	Active Not recruiting
Phase II, multi-center, open-label, single arm trial to evaluate the safety and efficacy of oral E7080 in medullary and iodine-131 refractory, unresectable differentiated thyroid cancers, stratified by histology. NCT00784303	Interventional open label non-randomized	This is a phase II study that aimed to investigate the safety and efficacy of oral E7080 (lenvatinib) in medullary and iodine-131 refractory, unresectable differentiated thyroid cancer.	Completed
Phase II study assessing the efficacy and safety of lenvatinib for anaplastic thyroid cancer (HOPE). NCT02726503	Interventional open label	This phase II study aims to investigate the efficacy and safety of lenvatinib for unresectable anaplastic thyroid cancer.	Recruiting
A multi-center, randomised, double-blind, placebo-controlled, phase III trial of lenvatinib (E7080) in I-131-refractory differentiated thyroid cancer in China. NCT02966093	Interventional double blind randomized	This phase III study primarily aims to compare progression-free survival of participants with radioiodine refractory differentiated thyroid cancer treated with lenvatinib or placebo, and secondarily to investigate adverse events.	Not yet recruiting
Post-marketing surveillance of Lenvima in Korean patients. NCT02764554	Observational prospective	This study aims to observe the safety profile of lenvatinib (Lenvima) in normal clinical practice.	Recruiting
Prospective, non-interventional, post-authorization safety study that includes all patients diagnosed as unresectable differentiated thyroid carcinoma and treated with sorafenib (JPMS-DTC). NCT02185560	Observational	Safety study that includes all patients diagnosed as unresectable differentiated thyroid carcinoma (DTC) and treated with sorafenib within a certain period.	Recruiting
Safety and efficacy of sorafenib in patients with advanced thyroid cancer: a Phase II clinical study. NCT02084732	Interventional	Describe the clinical activity and safety profile of sorafenib in the treatment of patients with advanced thyroid cancer (metastatic or recurrent) among a selected group of patients refractory to or ineligible to radioactive iodine (RAI) therapy.	Recruiting
Prospective, non-interventional, post-authorization safety study that includes all patients diagnosed as unresectable differentiated thyroid carcinoma and treated with sorafenib (JPMS-DTC). NCT02185560	Observational	This is a non-interventional, multi center post-authorization safety study that includes all patients diagnosed as unresectable differentiated thyroid carcinoma (DTC) and treated with sorafenib within a certain period.	Recruiting
Thyroid cancer and sunitinib (THYSU). NCT00510640	Interventional	The objective of the trial is to determine the objective tumor response rate (efficacy) in patients with locally advanced or metastatic anaplastic, differentiated or medullary thyroid carcinoma treated with sunitinib; a secondary objective is to evaluate the safety of sunitinib in these patients	Completed
Sutent adjunctive treatment of differentiated thyroid cancer (IIT Sutent). NCT00668811	Interventional	The primary objective is to assess clinical benefit rate, defined as complete response, partial response, or stable disease per RECIST criteria. The secondary objective will be to assess the safety of Sutent in this patient population.	Completed

**Table 3:** Overview of serious AE observed in clinical trials investigating TKI treatment; website used on 7 March 2017 [43].

Clinical Trial Title ID	Dose (mg/day)	# of Patients	Most Frequent Serious Adverse Effects	
SELECT: A multi center, randomized, double-blind, placebo-controlled, phase 3 trial of Lenvatinib (E7080) in ^131^I-refractory differentiated thyroid cancer. NCT01321554 [31,40]	24 Per os (PO)	261	4%	Pneumonia
			3%	Hypertension
			3%	Dehydration
			2%	Physical health deterioration
			2%	Renal failure
			2%	Pulmonary embolism
			2%	Sepsis
An open label phase I dose escalation study of E7080 (solid tumors). NCT00121719	0.1–32 PO	93	5%	Abdominal pain
			5%	Vomiting
			4%	Hypertension
			3%	Physical health deterioration
			3%	Pyrexia
Sorafenib in treating patients with advanced anaplastic thyroid cancer. NCT00126568 [46]	2 × 400 PO	20	15%	Disease progression
			10%	Death
			10%	Dyspnea
			5%	Thrombosis
			5%	Pulmonary disorders
Nexavar^®^ versus placebo in locally advanced/metastatic RAI-refractory differentiated thyroid cancer. NCT00984282 [24,25,26]	2 × 400 PO	207	5%	Secondary malignancy
			4%	Dyspnea
			4%	Musculoskeletal disorders
			3%	Pleural effusion
			2%	Fever
A continuation study using sunitinib malate for patients leaving treatment on a previous sunitinib study. NCT00428220	37.5 PO	223	5%	Disease progression
			4%	Abdominal pain
			3%	Vomiting
			3%	Diarrhea
			2%	Physical health deterioration
			2%	Pyrexia
			2%	Anemia
Sutent adjunctive treatment of differentiated thyroid cancer (IIT Sutent). NCT00668811 [45]	37.5 PO	23	13%	Hypertension
			13%	Leukopenia
			9%	Hand-foot syndrome
			9%	Anorexia
			4%	Neutropenia
			4%	Lymphopenia
			4%	Thrombocytopenia
			4%	Nausea
			4%	Gastrointestinal bleeding

**Table 4:** Different antihypertensive drugs for the management of TKI-induced hypertension.

Class	Drug	Dose	Recommendation
CCB Dihydropyridines	Amlodipine	2.5–10 mg/day	Great potency for reducing arterial smooth muscle cell contractility [39], effective therapy [49].
ACEi	Enalapril	Start with 5–20 mg/12–24 h, then max 40 mg/12–24 h	Particularly indicated in the setting of proteinuria [39], effective [49].
	Ramipril	Start with 2.5 mg/day, then 5 mg/day after 2 weeks, after another 2 weeks max 10 mg/day	
ARB	Losartan	50–100 mg/day	Particularly indicated in the setting of proteinuria [39], effective [49].
	Valsartan	80–320 mg/day	
	Irbesartan	150–300 mg/day	
BBA	Nebivolol	2.5–5 mg/day	Indicated for DTC; begin therapy of hypertension with a BBA [53].
Diuretics/Thiazides	Hydrochlorothiazide	Start with 12.5–25 mg/day, then 12.5 mg/day	Less-effective than CCB, ACEi or ARB [39], but often used [54].
Nitrate derivates	Long-acting nitrates: Isosorbide dinitrate (ISDN) or Isosorbide mononitrate (ISMN)	40–60 mg/day	Adequate response in hypertension refractory to ACEi and CCB [51].
α-blockers	Prazosin	2–20 mg/day	Used as additional therapy if BP is not sufficiently controlled.

CCB, calcium channel blockers; ACEi, Angiotensin converting enzyme inhibitors; ARB, angiotensin II receptor blockers; BBA, β-adrenoceptor antagonists; d, day.

## Abbreviations

AIIA	angiotensin II receptor antagonists
ACEi	angiotensin converting enzyme inhibitors
AE	adverse effects
AKT	protein kinase B
ATC	anaplastic thyroid cancer
CCB	calcium channel blockers
cGMP	cyclic guanosine monophosphate
DTC	differentiated thyroid cancer
EGF	endothelial growth factor
eNOS	endothelial nitric oxide synthase
ERK	extracellular signal regulated kinase
ET-1	endothelin-1
ET_A_	endothelin receptor type A
FGF	fibroblast growth factor
FGFR	fibroblast growth factor receptor
FTC	follicular thyroid cancer
MAPKK	mitogen activated protein kinase kinase
MTC	medullary thyroid cancer
NO	nitric oxide
PDGF	platelet derived growth factor
PDGFR	platelet derived growth factor receptor
PI3K	phosphatidylinositol-3-kinase
PDTC	poorly differentiated thyroid cancer
PO	per os
PTC	papillary thyroid cancer
RAI	radioactive iodine
RAS	rat sarcoma
RET	ret proto-oncogene
sGC	soluble guanylyl cyclase
TKI	tyrosine kinase inhibitor
VEGF	vascular endothelial growth factor
VEGFR	vascular endothelial growth factor receptor
